# Achieving robust and highly efficient nitrogen removal in a mainstream anammox reactor by introducing low concentrations of readily biodegradable organics

**DOI:** 10.3389/fmicb.2023.1186819

**Published:** 2023-04-28

**Authors:** Yandong Yang, Yanan Long, Jiarui Xu, Shichong Liu, Lei Liu, Changqing Liu, Yong Tian

**Affiliations:** ^1^School of Environmental and Municipal Engineering, Qingdao University of Technology, Qingdao, China; ^2^Engineering Research Center of Concrete Technology Under Marine Environment, Ministry of Education, Qingdao, China

**Keywords:** anammox, partial denitrification, mainstream, nitrogen removal, readily biodegradable organics

## Abstract

In this study, an anammox reactor was operated to treat low-strength (NH_4_^+^ + NO_2_^−^, 25–35  mg/L) wastewater without (phase I) or with (phase II) readily biodegradable chemical oxygen demand (rbCOD). In phase I, although efficient nitrogen removal was achieved at the beginning, nitrate accumulated in the effluent after long-term operation (75  days), resulting in a decrease in the nitrogen removal efficiency to 30%. Microbial analysis revealed that the abundance of anammox bacteria decreased from 2.15 to 1.78%, whereas that of nitrite-oxidizing bacteria (NOB) increased from 0.14 to 0.56%. In phase II, rbCOD, in terms of acetate, was introduced into the reactor with a carbon/nitrogen ratio of 0.9. The nitrate concentration in the effluent decreased within 2 days. Advanced nitrogen removal was achieved in the following operation, with an average effluent total nitrogen of 3.4 mg/L. Despite the introduction of rbCOD, anammox pathway still dominated to the nitrogen loss. High-throughput sequencing indicated that high anammox abundance (2.48%) further supports its dominant position. The improvement in nitrogen removal was attributed to the enhanced suppression of NOB activity, simultaneous nitrate polishing through partial denitrification and anammox, and promotion of sludge granulation. Overall, the introduction of low concentrations of rbCOD is a feasible strategy for achieving robust and efficient nitrogen removal in mainstream anammox reactors.

## 1. Introduction

Autotrophic nitrogen removal from municipal wastewater through partial nitritation/anammox (PN/A) has gained considerable attention over the past decade as it can bring an energy-neutral wastewater treatment plant (WWTP) (Kartal et al., 2010; Cao et al., 2017). Different process types based on the number of stages (i.e., single- or two-stage) have been developed to employ PN/A in WWTPs. The two-stage PN/A system can optimize the partial nitritation (PN) and anammox processes in two separate reactors, which favor the enrichment of different microorganisms (Isanta et al., 2015; Cao et al., 2017; Chen et al., 2020). Therefore, the two-stage PN/A process typically has a higher nitrogen removal rate than the single-stage PN/A process (Liu et al., 2018).

To date, the full-scale application of mainstream PN/As remains challenging. One of the major concerns is the high effluent nitrate concentration, which results in total nitrogen (TN) exceeding the discharge standard (Zhuang et al., 2022). In the two-stage PN/A process, nitrate can be generated both in the first-stage PN reactor owing to the activity of nitrite-oxidizing bacteria (NOB) and in the second-stage anammox reactor owing to the anammox and undesired NOB activities. According to the recent literature, nitrate production in the first-stage PN reactor can be minimized because of the development of various NOB suppression strategies, such as intermittent aeration, application of an ultra-low sludge retention time (SRT), and side-stream sludge treatment with inhibitors (Gilbert et al., 2014; Regmi et al., 2014; Wang et al., 2014, 2021). Whereas the second-stage anammox reactor became a major source of nitrate generation. Excessive nitrate production in anammox reactors due to undesired NOB activity has been reported in different reactor configurations, including sequencing batch reactors (SBR), completely stirred tank reactors, and expanded granular sludge blanket reactors (Sánchez Guillén et al., 2016; Liu et al., 2018; Zhang et al., 2019; Díaz et al., 2020; Li W. et al., 2020). Most of the NOB suppression methods developed in first-stage PN reactors are not applicable to second-stage anammox reactors because they may inhibit anammox activity. Therefore, strategies to reduce nitrate accumulation in anammox reactors must be investigated.

In early studies, readily biodegradable chemical oxygen demand (rbCOD) was considered a challenge for mainstream anammox processes, and hence, it should be eliminated by pretreatment as it can lead to the overgrowth of heterotrophs, which can outcompete anammox bacteria (Xu et al., 2015). However, recent studies have reported that the activities of anammox bacteria can be retained; in particular, nitrogen removal efficiencies can be improved to over 90% when anammox reactors were operated with an appropriate amount of rbCOD (C/N ratios of 0.5–1.5) (Li et al., 2016; Wang et al., 2019; Fu et al., 2021; Silveira et al., 2021; Chen et al., 2021a; He et al., 2023). However, most of these studies included wastewater treatment with high nitrogen concentrations (100–300 mg N/L), which can inhibit NOB growth due to the presences of free ammonia or free nitrous acid (Ge et al., 2015); consequently, excessive nitrate yield is negligible. It remains to be determined whether introducing rbCOD can resolve excessive nitrate production caused by NOB activity in mainstream anammox reactors. In addition, partial denitrification/anammox (PD/A) in anammox reactors can reduce the organic requirement for nitrogen removal and minimize the unfavorable effects of organics on anammox activity (Du et al., 2019). However, in most PD/A studies, ammonium and nitrate are the dominant nitrogen compounds in the influent (Du et al., 2019). In mainstream anammox reactors, nitrite is the main form of influent NO_x_^−^ and nitrate is gradually produced by the anammox reaction. The status of PD/A in mainstream anammox reactors in presence of rbCOD has rarely been reported and requires further investigation (Chen et al., 2020; Zhuang et al., 2022).

In this study, a mainstream anammox reactor was established and operated, with and without rbCOD, in two phases. The nitrogen removal performance, process stability, microbial community, and sludge morphology of the two phases were compared. Based on these data, the influence of low concentrations of rbCOD on the mainstream anammox process was assessed, and the potential of nitrogen removal enhancement with low concentrations of rbCOD was evaluated.

## 2. Materials and methods

### 2.1. Experimental setup

**Figure 1:**
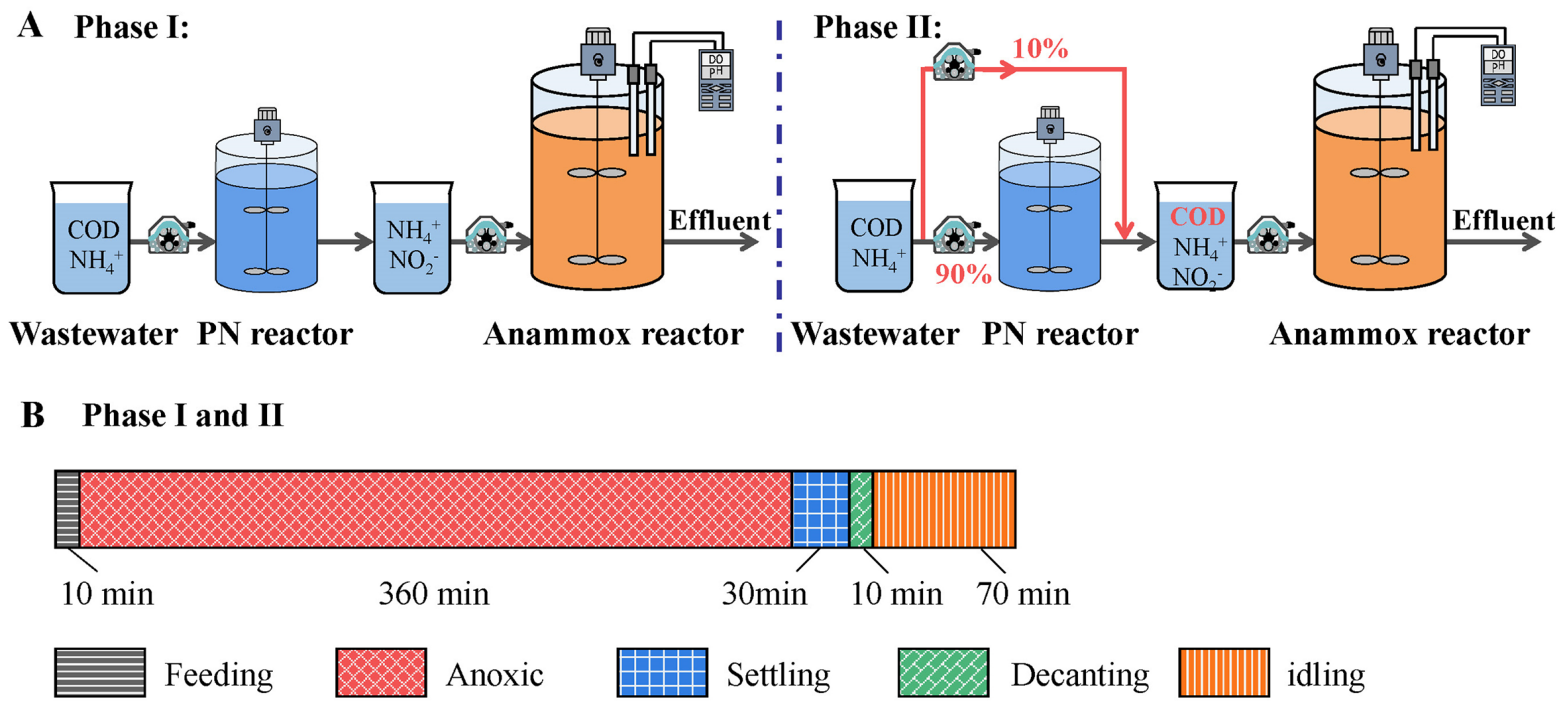
The schematic **(A)** and operational patterns **(B)** of the mainstream anammox reactor.

Mainstream anammox treatment was performed in a 10 L SBR (Figure 1), which was seeded with sludge collected from a lab-scale anammox reactor treating high nitrogen wastewater. After inoculation, the initial biomass concentration in the reactor was 4.9 g/L. The SBR was operated for three cycles per day. Each cycle consisted of 10 min of feeding (volume exchange ratio of 50%), 360 min of anoxic mixing, 30 min of settling, 10 min of discharging, and 70 min of idling (Figure 1B). The reactor was operated at room temperature of 24–26°C. Except for effluent biomass washout and sludge sampling, sludge was not disposed during the operation of the reactor. The SRT was estimated to be longer than 30 days.

Depending on the influent characteristics, the operation of the mainstream anammox reactor can be divided to two phases (Figure 1A and Table 1). In phase I (days 1–75), the mainstream anammox reactor was fed with the effluent (5 L) of a PN reactor that treated synthetic municipal wastewater. Description of the PN reactor has been available in Supplementary material. The feeding of the mainstream anammox reactor had low COD (25.1 ± 8.8 mg/L), most of which would likely be refractory organic compounds as the wastewater had been biotreated. In phase II (days 76–135), a portion of the raw synthetic municipal wastewater (0.5 L) was bypassed, and mixed with the PN effluent (4.5 L) in the middle tank. The mixed stream was then fed into the anammox reactor. Therefore, rbCOD was introduced into the system in the form of acetate (24.2 mg COD/L). Except for the differences in the influent organic composition, the operational patterns and conditions of the reactor were identical in phases I and II.

**Table 1:** Main characteristics of the influent of the mainstream anammox reactor.

	NH_4_^+^-N (mg/L)	NO_2_^−^-N (mg/L)	NO_3_^−^-N (mg/L)	TN (mg/L)	COD (mg/L)
Phase I	14.3 ± 2.5	14.1 ± 2.4	1.2 ± 0.8	29.4 ± 2.8	25.1 ± 8.8
Phase II	12.5 ± 1.8	12.3 ± 1.4	2.0 ± 0.6	26.8 ± 2.0	45.2 ± 11.2

### 2.2. Batch tests on the potential of PD/A in the reactor

Two *ex situ* batch tests were conducted to investigate the potential of the PD/A in the reactor. Sludge collected from the reactor was washed three times with deionized water, and then transferred to four separate 0.5-L conical flasks. After addition of organic and nitrogen substrates, the flasks were incubated in shaking incubators (200 rpm) at 25°C. During batch tests, the flasks were continuously stripped with nitrogen gas to provide an anoxic environment.

Batch Test I investigated whether nitrite could accumulate during denitrification. Therefore, only nitrate (NaNO_3_) and acetate were added to the flasks. The initial nitrate concentration was constant at 15 mg/L, and acetate was added at different dosages. The initial COD/NO_3_^−^-N ratios in four flasks were 0.5, 1.0, 1.5, and 2.0, respectively.

Batch Test II investigated whether denitrification could be integrated with the anammox process. In addition to nitrate (NaNO_3_) and acetate, ammonium (NH_4_Cl) was present in the feed. The initial ammonium and nitrate concentrations were 10 mg/L and 15 mg/L, respectively. While different dosages of acetate were added to four flasks, resulting in COD/NO_3_^−^-N ratios of 0.5, 1.0, 1.5, and 2.0, respectively.

### 2.3. Analytical methods

COD, NH_4_^+^-N, NO_2_^−^-N, NO_3_^−^-N, total nitrogen (TN), mixed liquor suspended solids, mixed liquor volatile suspended solids, and sludge volume index were measured using standard methods (APHA, 2005). The temperature, pH, and dissolved oxygen (DO) in the reactor were monitored on-line using a WTW Multi 3,620 instrument (WTW, Bavaria, Germany). Sludge size distribution was determined using a laser particle size analyzer (Malvern Mastersizer 2000, Malvern, United Kingdom).

### 2.4. Calculation of the nitrogen transformation pathways in typical cycles

Nitrogen transformation pathways in the reactor were computed based on the variations in nitrogen compounds concentrations in the typical cycles. The calculation only involves major nitrogen pathways. Different calculation methods are used in the two operating phases of the reactor. In phase I, the reactor was operated with negligible rbCOD input. Therefore, denitrification pathways were ignored in the calculation. Nitrogen conversions by anammox bacteria, ammonium-oxidizing bacteria (AOB) and NOB were calculated according to Eqs. 1–3.


(1) $$\begin{array}{l} {TN}_{\mathrm{anammox}}=\Delta N=({{NH}_{4}}^{+}-N_{\mathrm{initial}}+{{\mathrm{NO}}_{2}}^{-}-N_{\mathrm{initial}}\\ \;+{{\mathrm{NO}}_{3}}^{-}-N_{\mathrm{initial}})\;-({{NH}_{4}}^{+}-N_{\mathrm{final}}+{{\mathrm{NO}}_{2}}^{-}\\ \;-N_{\mathrm{final}}+{{\mathrm{NO}}_{3}}^{-}-N_{\mathrm{final}}) \end{array}$$

(2) $$\begin{array}{l} {{NH}_{4}}^{+}-N_{AOB}={{NH}_{4}}^{+}-N_{\mathrm{initial}}-{{NH}_{4}}^{+}\\ \;-N_{\mathrm{final}}-{TN}_{\mathrm{anammox}}/2.04 \end{array}$$

(3) $$\begin{array}{l} {{\mathrm{NO}}_{3}}^{-}-N_{NOB}={{\mathrm{NO}}_{3}}^{-}-N_{\mathrm{final}}-{{\mathrm{NO}}_{3}}^{-}\\ \;-N_{\mathrm{initial}}-{TN}_{\mathrm{anammox}}\times 0.26/2.04 \end{array}$$

Where ${TN}_{\mathrm{anammox}}$ represents TN removal by anammox bacteria, mg N/L; ${{NH}_{4}}^{+}-N_{AOB}$ represents ammonium consumption by AOB, mg N/L; ${{\mathrm{NO}}_{3}}^{-}-N_{NOB}$ represents nitrate production by NOB, mg N/L; ΔN is the difference between the bulk total inorganic nitrogen concentrations at the start and end of the cycle, mg N/L; ${{NH}_{4}}^{+}-N_{\mathrm{initial}}$ , ${{\mathrm{NO}}_{2}}^{-}-N_{\mathrm{initial}}$ , and ${{\mathrm{NO}}_{3}}^{-}-N_{\mathrm{initial}}$ are the bulk ammonium, nitrite, and nitrate concentrations, respectively, at the start of the cycle, mg N/L; ${{NH}_{4}}^{+}-N_{\mathrm{final}}$ , ${{\mathrm{NO}}_{2}}^{-}-N_{\mathrm{final}}$ , and ${{\mathrm{NO}}_{3}}^{-}-N_{\mathrm{final}}$ are the bulk ammonium, nitrite, and nitrate concentrations, respectively, at the end of the cycle, mg N/L.

In phase II, the reactor was operated under stricter anoxic condition than phase I. An overall nitrate removal was achieved (Section 3.2). Therefore, nitrification pathway was ignored in the calculation. Nitrogen conversions *via* anammox, denitratation (NO_3_^−^ → NO_2_^−^), and denitritation (NO_2_^−^ → N_2_) were calculated using Eqs. 4–6.


(4) $${TN}_{\mathrm{anammox}}=\left({{NH}_{4}}^{+}-N_{\mathrm{initial}}-{{NH}_{4}}^{+}-N_{\mathrm{final}}\right)\times \;2.04$$

(5) $$\begin{array}{l} {{\mathrm{NO}}_{3}}^{-}-N_{\mathrm{denitratation}}={{\mathrm{NO}}_{3}}^{-}-N_{\mathrm{initial}}+{TN}_{\mathrm{anammox}}\\ \;\times 0.26/2.04-{{\mathrm{NO}}_{3}}^{-}-N_{\mathrm{final}} \end{array}$$

(6) $${TN}_{\mathrm{denitritation}}=\Delta N-{TN}_{\mathrm{anammox}}$$

Where ${TN}_{\mathrm{anammox}}$ represents TN removal *via* anammox pathway, mg N/L; ${{\mathrm{NO}}_{3}}^{-}-N_{\mathrm{denitratation}}$ represents nitrate reduction *via* denitratation, mg N/L; ${TN}_{\mathrm{denitritation}}$ represents TN removal *via* denitritation, mg N/L.

### 2.5. High-throughput sequencing analysis

Sludge samples collected from the reactor on day 1, day 75 and day 134 were freeze-dried (BT2KXL, Virtis, United States) and then stored at −20°C. Genomic DNA was extracted using the Fast DNA Kit (MPBiomedicals, CA, United States) according to the manufacture’s instructions. The DNA concentration was measured using a Nanodrop Spectrophotometer (ND-1000, Isogen Life Science, Netherlands) to ensure the purity of the extract. The V3–V4 region of the 16S rRNA gene was amplified with bacterial primers 338F (5′-ACTCCTACG GGAGGCAGCAG-3′) and 806R (5′-GGACTAC HVGGGTWTCT AAT-3′). PCR products were sequenced on an Illumina MiSeq platform (Majorbio Bio-Pharm Technology Co., Ltd., Shanghai, China). Based on these data, the microbial composition was analyzed as described in our previous report (Yang et al., 2017). The raw reads have been deposited in the NCBI Sequence Read Archive (SRA) database (Accession Number: PRJNA942604).

## 3. Results

### 3.1. Nitrogen removal performance without and with rbCOD

**Figure 2:**
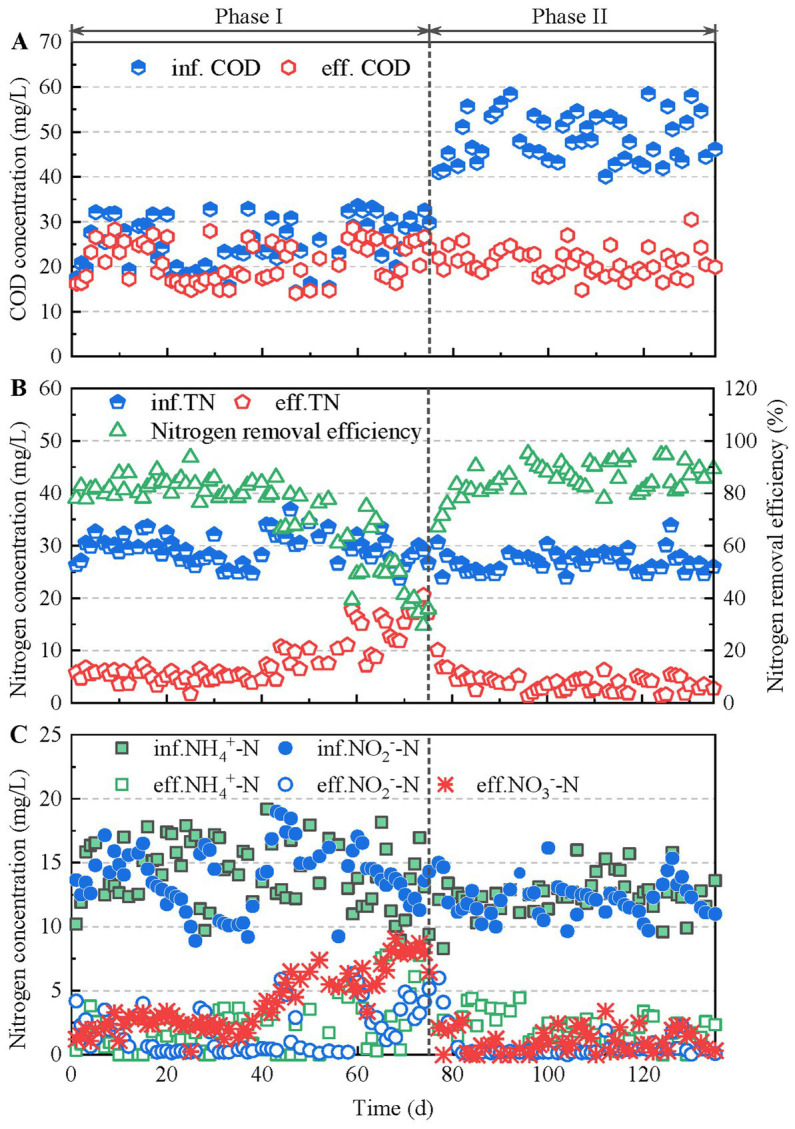
The COD **(A)** and TN **(B)** removal performance, as well as N-compound concentrations in the influent and effluent **(C)** of the mainstream anammox reactor.

In phase I, the mainstream anammox reactor was operated with negligible rbCOD input, as revealed by the insignificant COD removal (3.8 ± 2.1 mg/L) in the reactor (Figure 2A). Nitrogen removal performance was instable as shown in Figures 2B,C. Although good nitrogen removal performance was achieved at the beginning (days 1–40), the effluent nitrate concentration increased from 2.6 to 8.7 mg/L on day 41–75. In addition, ammonium and nitrite accumulated at the end of phase I, indicating a decline in the anammox activity in the reactor. Consequently, the nitrogen removal efficiency decreased from 83 to 30%.

In phase II, 24.2 mg COD/L acetate was introduced to the mainstream anammox reactor, increasing the rbCOD/TN ratio to 0.9. The average COD removal in the reactor was 27.7 mg/L, indicating a complete utilization of the rbCOD in the feeding (Figure 2A). Nitrate concentration in the effluent decreased from 8.7 mg/L to 2.1 mg/L within 2 days, and maintained at low level (1.1 ± 0.9 mg/L) in the following operation (Figure 2C). Overall, the nitrate removal was achieved. This suggested dentification in the reactor. In addition, the effluent ammonium and nitrite concentrations decreased simultaneously, indicating restoration of anammox activity. In phase II, advanced nitrogen removal was achieved with an average effluent TN of 3.4 mg/L and an average nitrogen removal efficiency of 87.2% (Figure 2B).

### 3.2. Changes in nitrogen transformation pathway after introducing rbCOD

**Figure 3:**
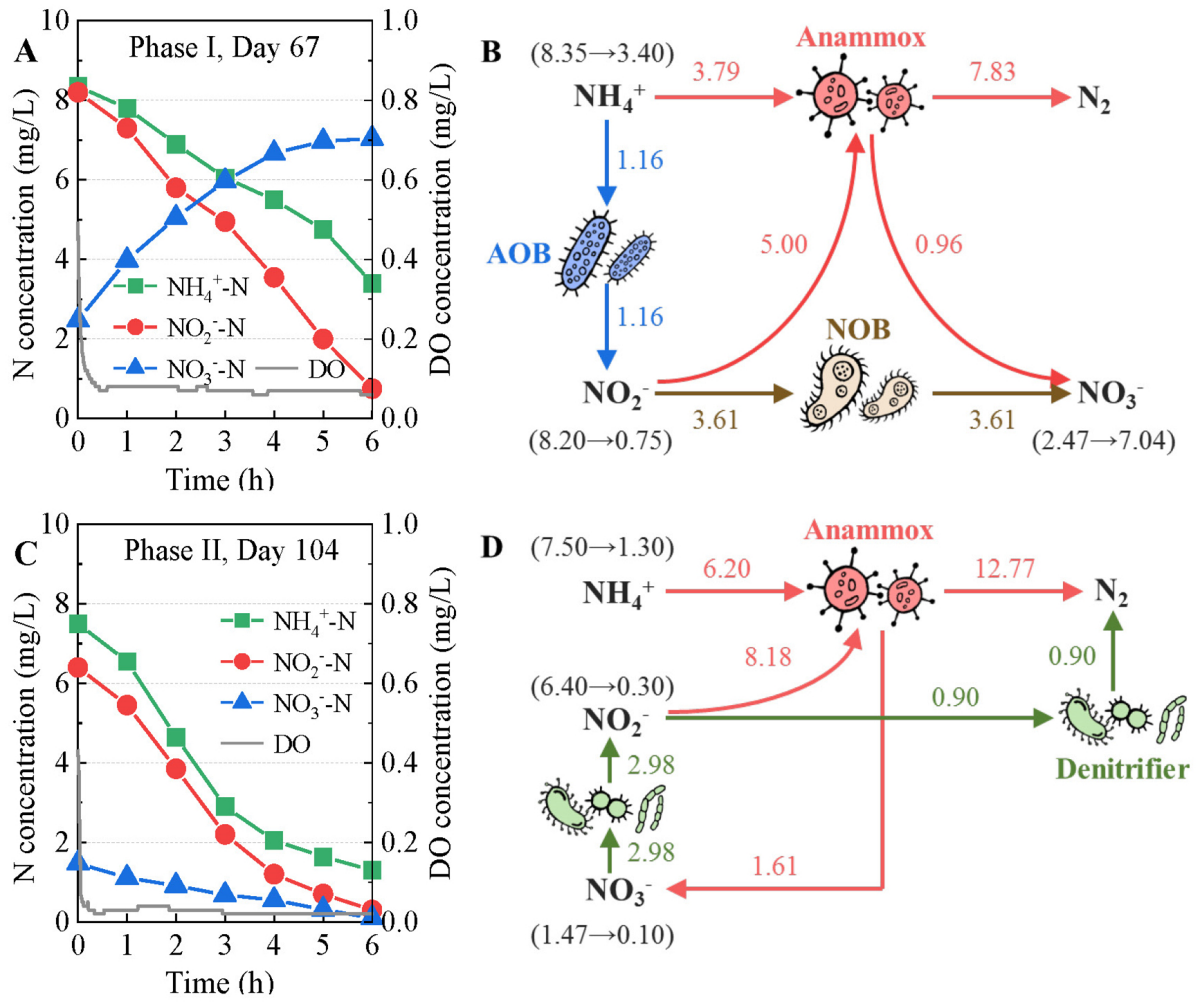
Variations in N-compound concentrations (**A**: phase I, **C**: phase II) and nitrogen transformation pathways (**B**: phase I, **D**: phase II) in the typical cycles of the mainstream anammox reactor.

The cycle analysis revealed changes in the nitrogen transformation pathway of the mainstream anammox reactor. In a typical phase I cycle (before introducing rbCOD), ammonium and nitrite were gradually removed, whereas nitrate accumulated considerably (Figure 3A). The ratio of NO_3_^−^-N produced over NH_4_^+^-N removed (ΔNO_3_^−^-N/ΔNH_4_^+^-N) was 0.92, which is much higher than the theoretical value of anammox reaction of 0.26 (Strous et al., 1998), indicating additional nitrate generation pathway. The nitrogen mass flow suggested that nitrification oxidized some ammonium to nitrite and oxidized part of the nitrite to nitrate in the anammox reactor (Figure 3B). Nitrification contributed to 79% of nitrate production, resulting in the insufficient nitrogen removal of the mainstream anammox reactor.

After introducing rbCOD in phase II, a simultaneous decrease in ammonium, nitrite and nitrate concentration was observed in a typical reaction cycle (Figure 3C). This indicates that not only the nitrate in the influent but also the nitrate generated by the anammox reaction were simultaneously removed by denitrification. Besides, nitrogen mass flow revealed partial denitrification (PD, NO_3_^−^ → NO_2_^−^) provided additional nitrite substrate for anammox (Figure 3D). As a result, *in situ* anammox activity was promoted. According to Figure 3D, anammox remained the dominant nitrogen removal pathway in the reactor with a contribution to nitrogen removal of 93%. Denitrification further improved the nitrogen removal efficiency, resulting in a low effluent TN concentration.

### 3.3. Potential of PD/A in anammox reactor revealed by batch tests

**Figure 4:**
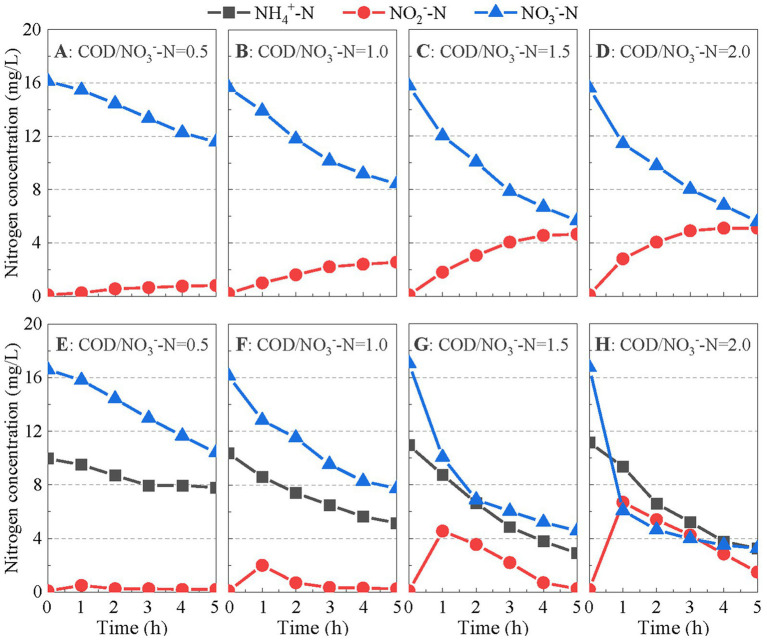
Variations in N-compound concentrations in Batch Tests I **(A–D)** and II **(E–H)**.

The potential of PD/A in the mainstream anammox reactor was further illustrated using *ex situ* batch tests. In Batch Test I, only nitrate and acetate were added to the feed to investigate denitrification. As shown in Figures 4A–D, nitrite accumulated at all COD/NO_3_^−^-N ratios. The highest nitrate-to-nitrite transformation ratio of 50.0% was obtained with a COD/NO_3_^−^-N ratio of 2.0. These results suggest that denitrification in the mainstream anammox reactor could provide additional nitrite for anammox.

In Batch Test II, ammonium was also added to the feed with nitrate and acetate. In the first hour, nitrate was removed significantly, whereas nitrite accumulated at all COD/NO_3_^−^-N ratios, indicating the occurrence of PD (Figures 4E–H). Thereafter, ammonium, nitrite, and nitrate concentrations simultaneously decreased. Under the strict anoxic conditions in batch test, ammonium was removed through anammox pathway. The nitrite substrate for the anammox reaction was generated through PD. Therefore, the potential of PD/A in the mainstream anammox reactor is proved.

### 3.4. Microbial evolution in the mainstream anammox reactor

**Figure 5:**
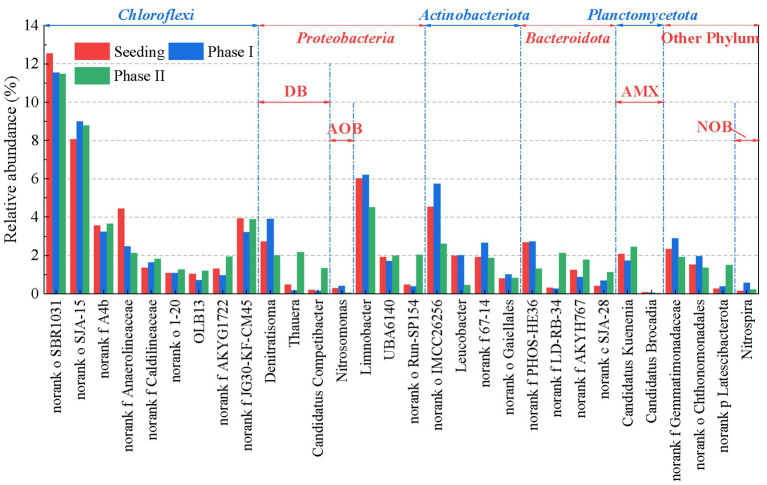
Variations in the microbial composition in the mainstream anammox reactor.

The effects of low-concentration rbCOD on the microbial community in the mainstream anammox reactor were investigated using high-throughput sequencing (Figure 5). In phase I (without rbCOD), the abundance of anammox bacteria (dominated by *Candidatus Kuenenia*) declined from 2.08 to 1.73%, while the abundances of AOB (*Nitrosomonas*) and NOB (*Nitrospira*) increased significantly from 0.29 to 0.40% and from 0.14 to 0.56%, respectively. These trends are consistent with the nitrate accumulation and nitrogen removal deterioration during reactor operation. After introducing rbCOD in phase II, the abundance of anammox bacteria was restored to 2.48%, whereas the abundances of AOB and NOB decreased to 0.05 and 0.23%, respectively. These results suggest that the proper introduction of rbCOD benefits the enrichment of anammox bacteria and the suppression of nitrifying bacteria. Additionally, a transition in the denitrifying bacteria population was observed after the introduction of rbCOD. *Denitratisoma* with the capacity of complete denitrification (NO_3_^−^ → N_2_) was the predominant denitrifying bacteria in phase I, and its abundance decreased from 3.91 to 2.00% in phase II. While *Thauera* and *Candidatus Competibacter*, which have been identified as the functional microorganisms for PD (Du et al., 2016; Shi et al., 2019; Ji et al., 2020), were considerably enriched in phase II with abundances increased from 0.18 to 2.15%, and from 0.15 to 1.35%, respectively. These results provided further evidence of PD/A in the reactor.

### 3.5. Granulation process in the mainstream anammox reactor

**Figure 6:**
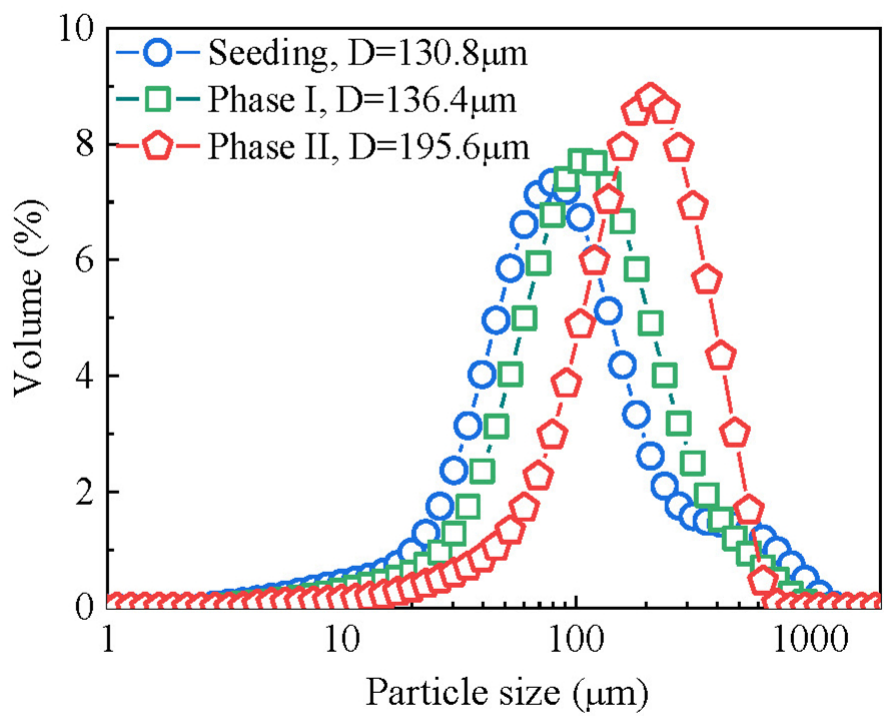
Variation in sludge size distribution in the mainstream anammox reactor.

The seeding sludge of the mainstream anammox reactor contained a specific quantity of granular sludge (D > 200 μm, 17.4%) and had an average diameter of 130.8 μm (Figure 6). The sludge size was stable in phase I and increased significantly to 195.6 μm in phase II. Granular sludge eventually constituted 52.5% of the total biomass in the reactor, indicating that granulation was promoted after the introduction of rbCOD in phase II. Granulation in the reactor can facilitate the enrichment of anammox bacteria because they are more prone to grow in large aggregates (Vlaeminck et al., 2010; Laureni et al., 2019).

## 4. Discussion

### 4.1. Mechanism of the nitrogen removal enhancement after introducing rbCOD

In the present study, the introduction of rbCOD quickly restored the mainstream anammox reactor from high nitrate accumulation (Figure 2). Advanced nitrogen removal was achieved in the presence of low concentrations of rbCOD. There may be multiple mechanisms behind the nitrogen removal enhancement in the mainstream anammox reactor after the introduction of rbCOD.

First, the suppression of nitrification was enhanced, which reduced *in situ* nitrate production. In phase I, the reactor was operated without rbCOD input. Relatively high nitrification activity results in an increase in nitrate production and a deterioration in nitrogen removal performance (Figures 2, 3), as demonstrated in the literature (Díaz et al., 2020; Li W. et al., 2020). The DO (0.06–0.08 mg/L) supporting nitrification transferred from influent and air at the top of the reactor during mechanical mixing (Figure 3A). Some nitrifier species, including complete ammonia oxidizer (comammox) and NOB in genus *Nitrospira*, exhibit high oxygen affinities (Liu and Wang, 2013; Akaboci et al., 2018; Roots et al., 2019). They may have contributed to nitrification in the reactor because the abundance of *Nitrospira* increased significantly (Figure 5). In contrast, the DO concentration in the reactor decreased to 0.02–0.04 mg/L in phase II after the introduction of rbCOD, possibly because of the activity of aerobic heterotrophic bacteria (Figure 3C). Therefore, stricter anoxic conditions were obtained in phase II than in phase I, which suppressed nitrification activity. Consequently, both AOB and NOB abundance decreased during phase II (Figure 5).

Second, PD/A was achieved in the reactor, which simultaneously removed the nitrate originating from the influent and produced by the anammox reaction. Batch tests have proved that nitrite can be accumulated during the denitrification of nitrate (Figures 4A–D). Besides, this part of nitrite can be utilized and removed by anammox bacteria with ammonium as the electron donor as revealed by cycle analysis (Figure 3D) and batch test (Figures 4E–H). In such ways, nitrate in the reactor was polished with a minor organic consumption, which improved the nitrogen removal efficiency of the reactor to a high level. In addition, although rbCOD was introduced with the influent at the beginning, nitrate was produced (*via* anammox) and reduced (*via* denitrification) continuously during the entire cycle (Figure 3). Therefore, endogenous denitrification was expected to occur. In this study, *Thauera* and *Candidatus Competibacter* were the functional bacteria responsible for PD during phase II (Figure 5). Both can restore rbCOD as endogenous carbon sources such as glycogen and polyhydroxyalkanoates (Lopez-Vazquez et al., 2009; Andreolli et al., 2022). Therefore, the rbCOD in the influent may have been transferred to endogenous carbon sources at the beginning and supported PD during the entire cycle.

Third, anammox activity and growth were promoted, which improves the process performance and stability. In this study, the direct effects of rbCOD (acetate) on anammox activity may be limited. Low concentrations of acetate does not inhibit anammox activity (Huang et al., 2014). Some anammox species, such as *Candidatus Anammoxoglobus propionicus* and *Candidatus Brocadia fulgida* can utilize short-chain fatty acids for energy generating (Kartal et al., 2007, 2008). However, this pathway may be insignificant in this study because anammox population in the reactor was dominated by genus *Candidatus Kuenenia* throughout the experiment. Therefore, rbCOD mainly promoted anammox activity in indirect ways. For example, the aerobic oxidation of rbCOD can consume the DO in bulk liquid and reduce its inhibitory effect on anammox activity (Strous et al., 1999). In addition, the anoxic utilization of rbCOD by PD of nitrate provided additional nitrite substrate for anammox bacteria and promoted anammox activity (Figure 3). Furthermore, the presence of rbCOD promoted extracellular polymers secretion and granulation in the mainstream anammox reactor (Figure 6) as demonstrated in the literature (Li et al., 2015; Chen et al., 2021a), which helped retain and enrich slow-growing anammox bacteria (Zhu et al., 2018; Chen et al., 2021b). Consequently, anammox abundance increased from 1.73 to 2.48% after introducing low concentrations of rbCOD (Figure 5). Meanwhile anammox pathway maintained its dominant position in nitrogen removal (Figure 3).

### 4.2. Application and further study

In this study, an effective and applicable strategy was employed to solve nitrate accumulation caused by undesired NOB activity in mainstream anammox reactors. Other strategies have been reported to reduce nitrate yield, including purging the oxygen in the influent with nitrogen gas, minimizing the surface area of the reactor in contact with the atmosphere by adopting a high height/diameter ratio, and providing protective covers in the anammox reactor (Díaz et al., 2020). However, these measures may not be economical or applicable in some cases, especially for upgrading existing WWTPs because they require major retrofitting of the reactor configuration. Hence, the present study included the strategy of introducing low concentration of rbCOD, which can reduce nitrate accumulation with a minor modification by redirecting part of the raw municipal wastewater to the anammox reactor or adding a small amount of external organic carbon (Le et al., 2019; Fofana et al., 2022). In addition, rbCOD can be provided using sludge fermentation liquid, if it is available in the WWTP (Cao et al., 2013; Basset et al., 2016; Ali et al., 2021). At last, it is noted that the proposed operating strategy for mainstream anammox process is also applicable to continuous flow reactors. However, additional measures may be required to enhance the retention of anammox bacteria, such as filling proper carriers and using cyclone to selective retain granular sludge (Wett et al., 2013; Yang et al., 2017).

Even when there is no excessive nitrate production by NOB, operating the mainstream anammox reactor with a low concentration of rbCOD is beneficial because of its potential to achieve complete nitrogen removal (Zhuang et al., 2022). Both PN/A and PD/A contributed to the nitrogen removal as reported in the literature (Li J. et al., 2020; Li et al., 2022). Specifically in this study, PN/A removed most of the nitrogen autotrophically, whereas PD/A removed the nitrate generated in the anammox reaction. A theoretical equation for this process is presented in Table 2 for Case III and compared with other anammox-based processes targeting complete nitrogen removal (Cases I and II). In Case I, PN/A removes nitrogen autotrophically, however 11% of ammonium is eventually converted to nitrate. The nitrate polishing through denitrification still consumes organic carbon. The overall organic requirement of Case I is 0.32 mg COD/mg N. In Case II, nitrite substrate for anammox is generated through the complete nitrification of ammonium and subsequent PD of nitrate. This approach requires additional oxygen and organic carbon than the nitrite generation pathway of PN. Overall, 2.35 mg O_2_/mg N and 0.73 mg COD/mg N are consumed in Case II. Case III has theoretical advantages over Cases I and II. First, nitrite is mainly generated through PN, therefore saving of oxygen and organic carbon is expected when compared with Case II. Second, nitrate polishing is achieved through PD/A, which consumes less organic carbon than Case I. In addition, the PD/A in Case III can remove a certain amount of ammonium without consuming oxygen, therefore oxygen demand can also be saved compared with Case I. Overall Case III can achieve 100% nitrogen removal with the least requirement for oxygen (1.76 mg O_2_/mg N) and organic matter (0.14 mg COD/mg N) in the three cases.

**Table 2:** Comparison of the theoretical oxygen and organic demand of different cases for complete nitrogen removal base on anammox.

	Reaction	Oxygen demand (mg O_2_/mg N)	Organic demand (mg COD/mg N)
Case I: PN/A + denitrification	Partial nitrification: NH_3_ + 1.5O_2_ → NO_2_^−^ + H_2_O + H^+^ (i) Anammox: NH_3_ + 1.32NO_2_^−^ + H^+^ → 1.02 N_2_ + 0.26NO_3_^−^ + 2H_2_O (ii) Denitrification: NO_3_^−^ + 0.625CH_3_COO^−^ + 0.625H^+^ → 0.5 N_2_ + HCO_3_^−^ + 0.25CO_2_ + 0.75H_2_O (iii) Total reaction by (i × 1.32 + ii)/2.32 + iii × 0.11: NH_3_ + 0.85O_2_ + 0.07CH_3_COO^−^ → 0.5 N_2_ + 0.11HCO_3_^−^ + 0.03CO_2_ + 1.51H_2_O + 0.07H^+^	1.94	0.32
Case II: Complete nitrification + PD/A	Complete nitrification: NH_3_ + 2O_2_ → NO_3_^−^ + H_2_O + H^+^ (i) Partial denitrification: NO_3_^−^ + 0.25CH_3_COO^−^ → NO_2_^−^ + 0.25HCO_3_^−^ + 0.25CO_2_ + 0.25H_2_O (ii) Anammox: NH_3_ + 1.32NO_2_^−^ + H^+^ → 1.02 N_2_ + 0.26NO_3_^−^ + 2H_2_O (iii) Total reaction by ((ii × 1.32 + iii) + i × 1.06)/2.06: NH_3_ + 1.03O_2_ + 0.16CH_3_COO^−^ → 0.5 N_2_ + 0.16HCO_3_^−^ + 0.16CO_2_ + 1.65H_2_O + 0.03H^+^	2.35	0.73
Case III PN/A + PD/A	Partial nitrification: NH_3_ + 1.5O_2_ → NO_2_^−^ + H_2_O + H^+^ (i) Anammox: NH_3_ + 1.32NO_2_^−^ + H^+^ → 1.02 N_2_ + 0.26NO_3_^−^ + 2H_2_O (ii) Partial denitrification: NO_3_^−^ + 0.25CH_3_COO^−^ → NO_2_^−^ + 0.25HCO_3_^−^ + 0.25CO_2_ + 0.25H_2_O (iii) Total reaction by ((iii × 0.26 + ii) + i × 1.06)/2.06: NH_3_ + 0.77O_2_ + 0.03CH_3_COO^−^ → 0.5 N_2_ + 0.03HCO_3_^−^ + 0.03CO_2_ + 1.52H_2_O + 0.03H^+^	1.76	0.14

Despite these theoretical and practical advantages, several issues need to be resolved before employing the proposed nitrate control strategy. First, nitrogen removal mechanism should be further investigated. In this study, some secondary nitrogen metabolism pathways were ignored when computing the nitrogen transformation in the reactor, which might have an impact on the accuracy of the calculation. The complex microbial nitrogen-recycling networks should be resolved in future studies using advanced technologies such as isotope labeling technique, metagenomics and metatranscriptomics (Wang et al., 2019; Yang et al., 2020). Second, the feasibility of the nitrate control strategy should be further verified using real municipal wastewater, although PD and PD/A using complex organic carbons in real municipal wastewater have been reported in the literature (Shi et al., 2019; Du et al., 2020; Ji et al., 2020). Besides, prolonged experiments are required to investigate the long-term effects of organic additions, particularly with seasonal temperature variations. Third, the optimal ranges of the rbCOD concentration and rbCOD/TN ratio should be determined in future studies to minimize organic consumption in real applications.

## 5. Conclusion

In this study, a mainstream anammox treatment was established in an SBR. When the reactor was operated with no rbCOD, high levels of nitrate accumulated in the effluent because of undesired NOB activity, resulting in reduced nitrogen removal efficiency. In contrast, operation with a low rbCOD concentration restored the anammox reactor from nitrate accumulation and achieved highly efficient nitrogen removal. At a low concentration of rbCOD, anammox remained the dominant nitrogen removal pathway, whereas PD/A removed the nitrate generated by the anammox reaction. Besides, NOB were effectively suppressed because of the stricter anoxic environment. In addition, granulation was promoted, which favored the enrichment of slow-growing anammox bacteria. Overall, the introduction of low concentrations of rbCOD is a feasible strategy for achieving robust and efficient nitrogen removal in mainstream anammox reactors.

## Data availability statement

The datasets presented in this study can be found in online repositories. The names of the repository/repositories and accession number(s) can be found in the article/Supplementary material.

## Author contributions

YY: funding acquisition, conceptualization, data curation, and writing—original draft. YL: formal analysis, validation, investigation, and writing—original draft. JX: formal analysis and data curation. SL: methodology and resources. LL: software and data curation. CL: project administration and writing—review and editing. YT: funding acquisition and writing—review and editing. All authors contributed to the article and approved the submitted version.

## Funding

This research was financially supported by the Natural Science Foundation of Shandong Province, China (Grant No. ZR2019BEE070), the National Natural Science Foundation of China (Grant No. 22176106), the Shandong Province Higher Educational Program for Introduction and Cultivation of Young Innovative Talents (2021), and the Open Research Fund of Engineering Research Center of Concrete Technology under Marine Environment, Ministry of Education (Grant No. TMduracon2022041).

## Conflict of interest

The authors declare that the research was conducted in the absence of any commercial or financial relationships that could be construed as a potential conflict of interest.

## Publisher’s note

All claims expressed in this article are solely those of the authors and do not necessarily represent those of their affiliated organizations, or those of the publisher, the editors and the reviewers. Any product that may be evaluated in this article, or claim that may be made by its manufacturer, is not guaranteed or endorsed by the publisher.
