# Supplementary material for: Achieving robust and highly efficient nitrogen removal in a mainstream anammox reactor by introducing low concentrations of readily biodegradable organics
Source: Front Microbiol. 2023 Apr 28;14:1186819. doi: 10.3389/fmicb.2023.1186819 (PMC10175599; doi:10.3389/fmicb.2023.1186819)
Supplement: Supplementary file 1 [file presentation_1.pdf]

Supplementary Material

Achieving robust and highly efficient nitrogen removal in a mainstream anammox reactor by introducing low concentrations of readily biodegradable organics

Yandong Yang*, Yanan Long, Jiarui Xu, Shichong Liu, Lei Liu, Changqing Liu, Yong Tian

*** Correspondence:** Yandong Yang: yangyandong@qut.edu.cn

1 Operation and performance of the partial nitritation (PN) reactor

1.1 Composition of the synthetic municipal wastewater

In this study, the mainstream anammox reactor was fed with the effluent of a PN reactor that treated synthetic municipal wastewater. The synthetic municipal wastewater used in the study consisted of (per liter tap water): 0.320 g sodium acetate (chemical oxygen demand (COD) = 300 mg/L), 0.191 g NH_4Cl (NH_4^+-N = 50 mg/L), 0.022 g KH_2PO_4 (PO_4^{3-}-P = 5 mg/L), 0.030 g $MgSO_4$, 0.450 g $NaHCO_3$, 0.020 g $CaCl_2$ and 1 mL trace elements solution. The composition of the trace elements solution has been described by Ni et al. (2009).

1.2 Operation of the PN reactor

Mainstream PN was achieved in a lab-scale sequencing batch reactor with a working volume of 10 L. The reactor was inoculated with sludge collected from another PN reactor treating ammonium-rich wastewater. The PN reactor was operated for six cycles per day. Each cycle consisted of 10 min of feeding (volume exchange ratio of 50%), 40 min of anoxic mixing, 90–150 min of aeration, 20 min of settling, 10 min of discharging, and 10–70 min of idling. The dissolved oxygen concentration during aeration period was higher than 6 mg/L. The reactor was operated at room temperature of 24–26 °C. A ultra-low sludge retention time of 3–5 days was adopted for NOB out-selection (Regmi et al., 2014; Wang et al., 2021).

1.3 Performance of the PN reactor

A high COD removal efficiency of COD 89.6±2.3% was achieved in the PN reactor. The residual COD concentration was 24.9 mg/L, most of which would be refractory organic compounds, such as cell decay materials and soluble microbial products. Nitritation was stable during the entire experiment by controlling a short SRT (3–5 days). Effluent nitrate concentrations were 1.5±0.8 mg/L. Nitrite accumulation ratio was as high as 90.0±4.7%. Mainstream PN was achieved in a lab-scale sequencing batch reactor with a working volume of 10 L.

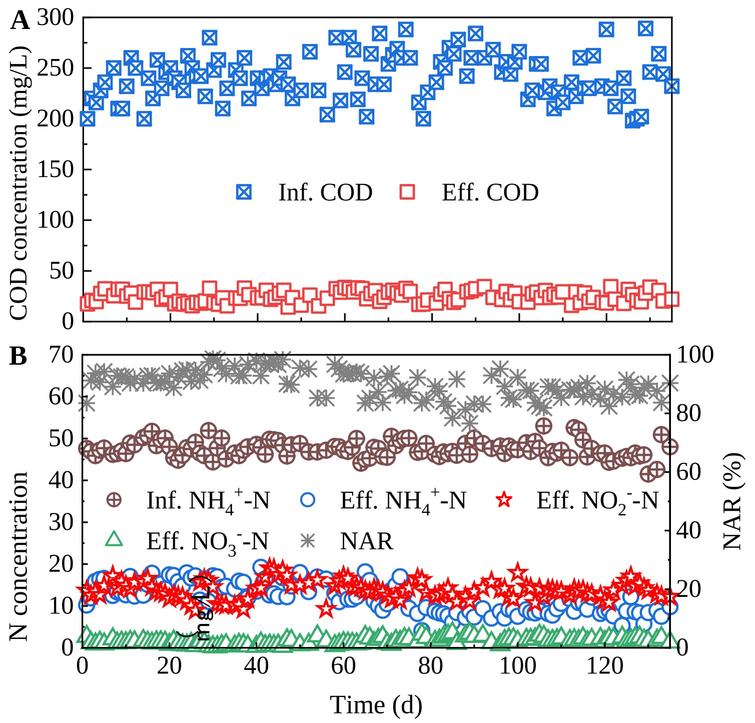

Supplementary Figure 1. The COD removal performance (A), influent ammonium and effluent ammonium, nitrite, nitrate, and nitrite accumulation ratio (NAR, NO_2^--N/(NO_2^--N+NO_3^--N)) (B) of the PN reactor.

References

Ni, B.-J., Fang, F., Xie, W.-M., Sun, M., Sheng, G.-P., Li, W.-H., et al. (2009). Characterization of extracellular polymeric substances produced by mixed microorganisms in activated sludge with gel-permeating chromatography, excitation–emission matrix fluorescence spectroscopy measurement and kinetic modeling. *Water Res.* 43, 1350–1358. doi: 10.1016/j.watres.2008.12.004.

Regmi, P., Miller, M.W., Holgate, B., Bunce, R., Park, H., Chandran, K., et al. (2014). Control of aeration, aerobic SRT and COD input for mainstream nitritation/denitritation. *Water Res.* 57, 162–171. doi: 10.1016/j.watres.2014.03.035.

Wang, Z., Peng, Y., Li, J., Liu, J., Zhang, Q., Li, X., et al. (2021). Rapid initiation and stable maintenance of municipal wastewater nitritation during the continuous flow anaerobic/oxic process with an ultra-low sludge retention time. *Water Res.* 197:117091. doi: 10.1016/j.watres.2021.117091.
